# Evaluation of Hyaluronic Acid to Modulate Oral Squamous Cell Carcinoma Growth In Vitro

**DOI:** 10.3390/jfb11040072

**Published:** 2020-10-01

**Authors:** Jordan Ringer, Bryan Morrison, Karl Kingsley

**Affiliations:** 1 Department of Clinical Sciences, University of Nevada, Las Vegas—School of Dental Medicine, 1001 Shadow Lane, Las Vegas, NV 89106, USA; ringer@unlv.nevada.edu; 2 Department of Biomedical Sciences and Director of Student Research, University of Nevada, Las Vegas—School of Dental Medicine, 1001 Shadow Lane, Las Vegas, NV 89106, USA; morrib4@unlv.nevada.edu

**Keywords:** oral cancer, hyaluronic acid, chemotherapy, paclitaxel

## Abstract

Introduction: Previous studies have demonstrated that glycosaminoglycan hyaluronic acid (HA) is capable of mediating oral tumor growth. Some clinical evidence has suggested reduced HA expression predicts poor cancer prognosis and that HA-chemotherapy conjugates may function synergistically to inhibit oral tumor growth. Other studies have found conflicting results that suggest enhanced CD44-HA-mediated growth and proliferation. Due to the lack of clarity regarding HA function, the primary goal of this study was to investigate the effects of HA using well-characterized oral cancer cell lines. Methods: Using several commercially available oral squamous cell carcinoma lines (and a normal non-cancerous control), 96-well growth and viability assays were conducted using HA (alone and in combination with chemotherapeutic agents paclitaxel and PD98059). Results: Different results were observed in each of the cell lines evaluated. HA induced small, non-significant changes in cellular viability among each of the cell lines within a narrow range (1–8%), *p* = 0.207. However, HA induced differing effects on growth, with minimal, non-significant changes among some cell lines, such as SCC4 (+1.7%), CCL-30 (−2.8%), and SCC15 (−2.5%), *p* = 0.211 and more robust inhibition among other cell lines, SCC9 (−24.4%), SCC25 (−36.6%), and CAL27 (−47.8%), *p* = 0.0001. Differing effects were also observed with growth and viability under concomitant administration of HA with PD98059 or paclitaxel. Further analysis of these data revealed strong inverse (Pearson’s) correlations between initial baseline growth rate and responsiveness to HA administration, ranging from R = −0.27 to R = −0.883. Conclusion: The results of this study revealed differing responses to HA, which may be inversely correlated with intrinsic characteristics, such as the baseline growth rate. This may suggest that the more rapidly growing cell lines are more responsive to combination therapy with hyaluronic acid; an important finding that may provide insights into the mechanisms responsible for these observations.

## 1. Introduction

Previous studies have demonstrated that the glycosaminoglycan hyaluronic acid (HA) is capable of mediating oral tumor growth [1,2,3]. For example, recent studies of liver and pancreatic cancers have demonstrated the role of the tumor microenvironment and HA, more specifically, to modulate growth and progression [4,5]. This has led to an increased need to understand not only the phenotypic-modulating potential of HA but also the interactive effects of chemotherapeutic-based treatments involving HA co-administration [6,7].

Some evidence from clinical studies has suggested reduced HA expression may be sufficient to provide diagnostic and prognostic information for different types of cancer, including breast and bladder cancers [8,9,10]. More recent evidence has suggested the prognostic and therapeutic implications of HA expression may also be useful in some subsets of oral cancers [11,12]. For example, in HPV-negative tumors, these studies revealed that high levels of stromal HA expression were, in fact, predictors of poor clinical outcomes and reduced survival, which may be a function of interactions with the extracellular matrix (ECM) and HA, in particular [1,11]. For example, some evidence now suggests that degradation of HA by hyaluronidase (HAase) in some head and neck tumors may function to create smaller fragments capable of stimulating angiogenesis—a strong negative indicator of survival and outcome among oral cancer patients [13,14].

Despite these findings, much remains to be discovered regarding the therapeutic potential of HA for oral cancer treatment. For example, some evidence has suggested improved clinical outcomes for chemotherapeutic delivery with HA [15], while others have found more complicated and conflicting results [16]. This evidence may suggest that combination therapies with HA might facilitate chemotherapeutic potential, although much remains to be evaluated regarding which patients and specific clinical pathophysiologic indications are most appropriate for the use and administration of HA [17,18]. Based upon the conflicting nature of studies in this area, the primary goal of this study was to investigate the effects of HA (alone and in combination with chemotherapeutic agents) using well-characterized oral cancer cell lines.

## 2. Methods

### 2.1. Cell Culture

Several commercially available cell lines were obtained from the American Type Culture Collection (ATCC; Manassas, VA, USA), which included the oral squamous cell carcinoma (SCC) lines SCC4 (CRL-1624), SCC9 (CRL-1629), SCC15 (CRL-1623), SCC25 (CRL-1628), and CAL27 (CRL-2095). The nasal septum carcinoma line CCL-30 (RMPI-2650) and normal non-cancerous oral gingival cells HGF-1 (CRL-2014) were also obtained (Table 1).

Available cell lines were verified and cross-checked against the International Cell Line Authentication Committee (ICLAC) database to ensure these were not among the currently known cross-contaminated or misidentified cell lines. Short tandem repeat (STR) profiling results for these cell lines were compared using the eight STR loci from the ATCC database for cell line authentication.

In brief, cells were thawed and cultured according to the recommended protocol from the supplier (ATCC). CAL27, CCL-30, and HGF-1 cells were cultured in Dulbecco’s modified Eagle’s medium (DMEM) modified to contain 10% fetal bovine serum (FBS) and 1% penicillin–streptomycin. The other oral cancer cell lines (SCC4, SC9, SCC15, SCC25) were cultured in a 1:1 mixture of Dulbecco’s modified Eagle’s medium and Ham’s F12 medium containing 1.2 g/L sodium bicarbonate, 2.5 mM L-glutamine, 15 mM 4-(2-hydroxyethyl)-1-piperazineethanesulfonic acid (HEPE)S and 0.5 mM sodium pyruvate. All cells were maintained in a humidified biosafety level-2 (BSL-2) tissue culture chamber. Experiments were performed with cells between passages five and passage ten.

### 2.2. Reagents

Hyaluronic acid (HA) was obtained from MP Biomedicals (ThermoFisher Scientific; Fair Lawn, NJ, USA, CAS 9004-61-9) with a molecular weight (MW) of 776.651 g/mol. One chemotherapeutic agent used in other HA-combination therapies studies of head and neck cancers [17], paclitaxel (PTX) or Taxol, was obtained from ACROS Organics (ThermoFisher Scientific, CAS 33069-62-4) with an MW of 853.9 g/mol. A positive control, the cellular mitogen-activated protein kinase (MAP) kinase (MEK) inhibitor PD98059, was obtained from Invitrogen (ThermoFisher Scientific, Catalog number PHZ1164) with an MW of 267.3 Daltons (Da).

### 2.3. Cellular Viability

The viability of cells was determined at the beginning and end of each experimental assay using the trypan blue exclusion assay. In brief, a trypan blue 0.4% solution (Gibco) was added to control and experimental cells to create a 1:1 dilution, which was incubated at room temperature. Each plate was placed on a hemocytometer grid on a Zeiss Axiovert inverted microscope (Carl Zeiss AG, Oberkochen, Germany). Four to six grids (1.0 mm^2^) were counted and averaged to determine cell number. To verify these results, control and experimental cells were also trypsinized and diluted 1:1 with trypan blue for viability using a BioRad TC20 automated cell counter (Bio-Rad Laboratories, Hercules, CA, USA).

### 2.4. Proliferation Assays

Cells were plated in 96-well tissue culture plates at a density of 1.2 × 10^5^ cells/mL and allowed to grow for 24, 48, and 72 h. Experimental cells were treated with 10 ng/mL of PD98059, paclitaxel (Taxol), or both in the presence or absence of hyaluronic acid (10 ng/well), which were administered concomitantly at the beginning of each experimental trial. Cells were subsequently fixed with 10% formalin and stained using Gentian violet. Absorbance readings for each plate were measured using a BioTek ELx808 (BioTek, Winooski, VT, USA) microplate reader at 595 nm to approximate cell number and confluence.

### 2.5. Statistical Analysis

Differences between continuous variables (absorbance readings) were calculated using parametric statistical analysis methods, including two-tailed Student’s *t*-tests and an alpha level of 0.05 to determine significance. All experiments were performed in triplicate with *n* = 8 from each experimental trial for a total *n* = 24 for all experimental conditions.

## 3. Results

Cell cultures were established for each cell line, and viability was determined to establish the baseline before experimentation (Table 2). In brief, the viability of all cell lines ranged from 74% to 93%—with the highest viability observed among the oral squamous cell carcinomas. For example, SCC25, SCC15, SCC9, and CAL27 exhibited viability of 93%, 91%, 89%, and 89%, respectively. One additional oral cancer cell line (SCC4) and the nasal carcinoma (CCL-30) exhibited slightly lower viability (82% and 74%, respectively). The normal human gingival fibroblast cell line HGF-1 exhibited a viability of 88%.

**Figure 1:**
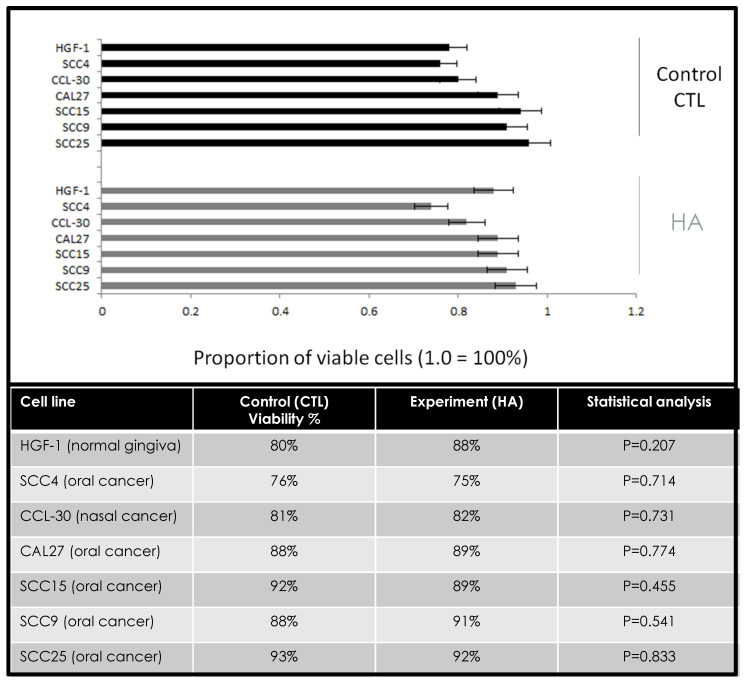
Effects of hyaluronic acid (HA) on cellular viability. Comparison of control and experimental (HYAL) assays demonstrated small, non-significant changes in cellular viability among each of the cell lines within a narrow range over 72 h (1–8%), *p* = 0.207 at 72 h (24, 48 h data not depicted).

To determine any effects of hyaluronic acid on survival, cells were plated in 96-well assays, and average viability was measured each day over three days (Figure 1). These data revealed that no significant differences were found in cellular viability between the control and experimental cell lines, *p* = 0.207. More specifically, a few cell lines exhibited small but not significant increases in viability, including HGF-1 (+8%), SCC9 (+3%), CCL-20 (+1%), and CAL27 (+1%), while others exhibited slight decreases in viability (SCC4, −1%; SCC15, −3%, SCC25, −1%).

**Figure 2:**
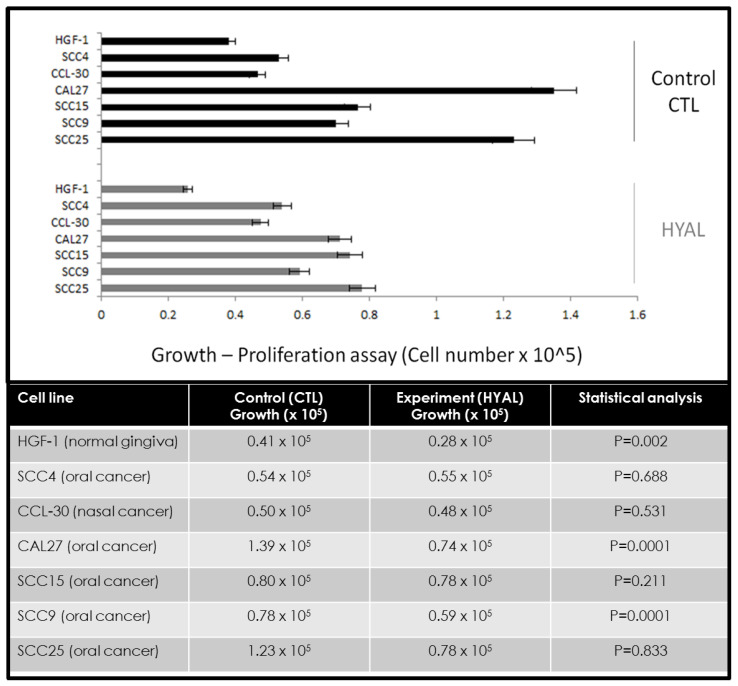
Effects of hyaluronic acid (HA) on cellular growth. Comparison of control and experimental (HYAL) assays demonstrated different effects, with minimal, non-significant changes among some cell lines, such as SCC4 (+1.7%), CCL-30 (−2.8%), and SCC15 (−2.5%), *p* = 0.211 at 72 h (24, 48 h data not depicted). More robust inhibition was observed among other cell lines, including SCC9 (−24.4%), SCC25 (−36.6%), and CAL27 (−47.8%), *p* = 0.0001. Growth inhibition was also observed among the normal, non-cancerous cell line HGF-1 (−31.8%), *p* = 0.002.

Following each measurement of viability, cells were also processed to measure growth to determine any effects of hyaluronic acid on proliferation (Figure 2). These data revealed different effects on growth that did not correlate with the effects on cellular viability. For example, some cells did not exhibit any significant changes to cellular growth between the control and experimental (HA) assays, including SCC4 (+1.7%), CCL-30 (−2.8%), and SCC15 (−2.5%), *p* = 0.211. However, some oral cancer cell lines exhibited robust reductions in cellular growth under the experimental conditions, such as SCC9 (−24.4%), SCC25 (−36.6%), and CAL27 (−47.8%), *p* = 0.0001. Interestingly, the normal oral gingiva cell line HGF-1 also demonstrated a reduction in growth (−31.8%), *p* = 0.002.

**Figure 3:**
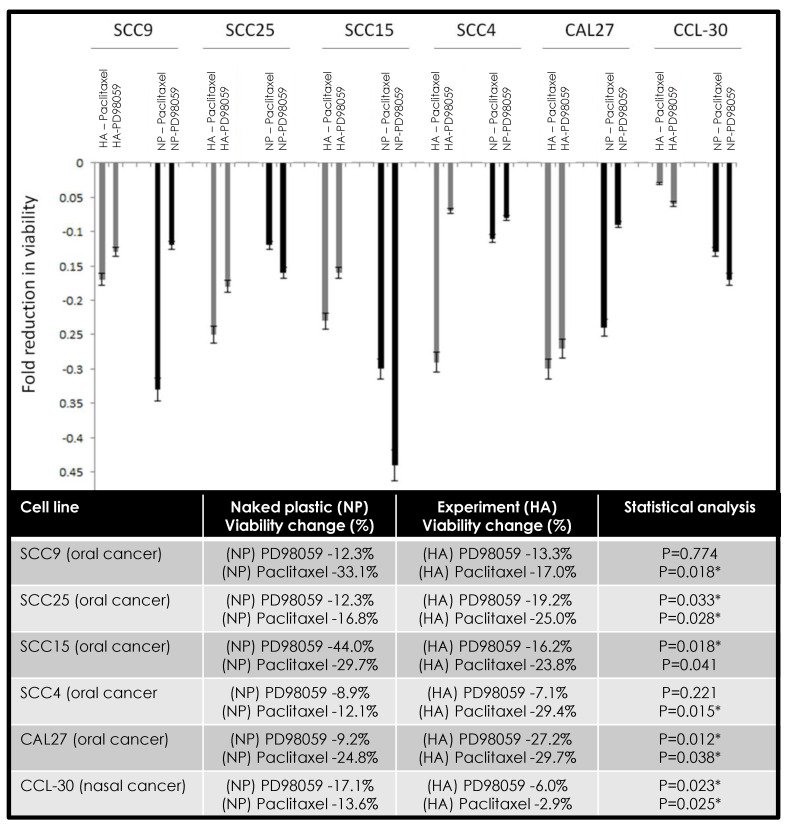
Combined effects of hyaluronic acid (HA) and PD98059 or paclitaxel on cellular viability. Differing effects were observed among cell lines under combination therapy with some cells exhibiting more robust reductions in cell viability under HA and PD98059 (SCC9, SCC25, CAL27) or HA and paclitaxel (SCC25, SCC4, CAL27). Some increases in viability were observed under HA and PD98059 with SCC15, SCC4, and CCL-30 cells, with increases also observed with HA and paclitaxel among SCC9, SCC15, and CCL-30 cells. Note: * indicates statistical significance *p* < 0.05.

The combination of chemotherapeutic agents alone or in combination with hyaluronic acid demonstrated different effects in modulating the viability among various oral cancer cell lines (Figure 3). More specifically, the combination of HA and PD98059 reduced cellular viability in SCC9 (−1.0%), SCC25 (−6.9%), and CAL27 (−18.0%) cells compared with naked plastic (NP) and PD98059, while HA and paclitaxel reduced viability in SCC25 (−8.2%), SCC4 (−17.3%), and CAL27 (−4.9%) cells. However, in other cells, the combination of HA and PD98059 increased cellular viability, such as SCC15 (+16.1%), SCC4 (+1.8%), and CCL-30 (+11.1%), compared with NP and PD98058, while the combination of HA and paclitaxel increased viability among SCC9 (+16.1%), SCC15 (+5.9%), and CCL-30 (+10.7%).

**Figure 4:**
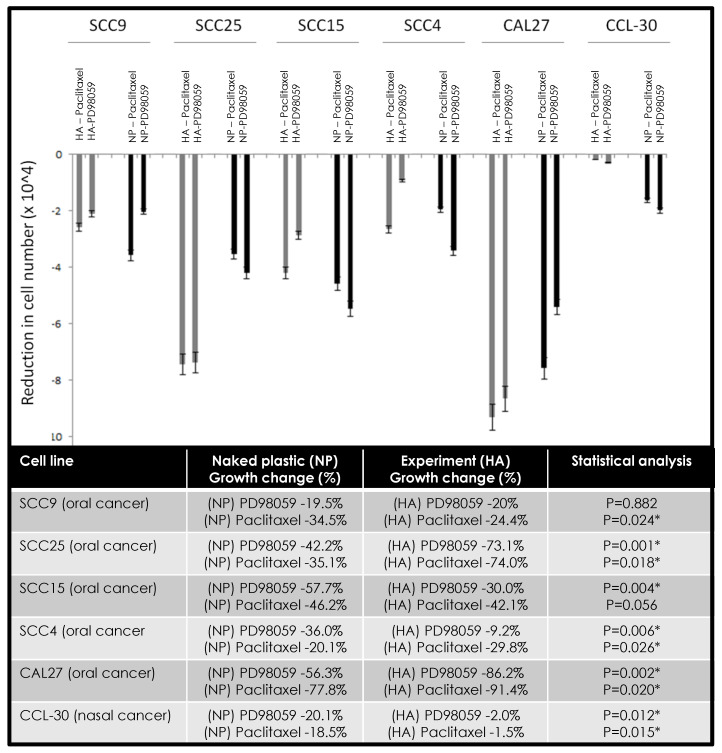
Effects of hyaluronic acid (HA) and PD98059 or paclitaxel on cellular growth. Differing effects were observed among cell lines under combination therapy with some cells exhibiting more robust reductions in cell proliferation under HA and PD98059 (SCC9, SCC25, CAL27) or HA and paclitaxel (SCC25, SCC4, CAL27). Some increases in growth were observed under HA and PD98059 with SCC15, SCC4, and CCL-30 cells, with increases also observed with HA and paclitaxel among SCC9, SCC15, and CCL-30 cells. Note: * indicates statistical significance *p* < 0.05.

The combination of chemotherapeutic agents alone or in combination with hyaluronic acid demonstrated different effects in modulating proliferation and growth among various oral cancer cell lines (Figure 4). More specifically, the combination of HA and PD98059 reduced cellular growth in SCC9 (−0.5%), SCC25 (−30.9%), and CAL27 (−29.9%) cells compared with NP and PD98059, while HA and paclitaxel reduced viability in SCC25 (−38.9%), SCC4 (−9.7%), and CAL27 (−13.6%) cells. However, in other cells, the combination of HA and PD98059 increased cellular growth, such as SCC15 (+27.7%), SCC4 (+26.8%), and CCL-30 (+18.1%) compared with NP and PD98058, while the combination of HA and paclitaxel increased growth among SCC9 (+10.1%), SCC15 (+4.1%), and CCL-30 (+17.0%).

Due to the differing nature of cellular phenotype responsiveness observed in these studies, the changes in growth and viability to hyaluronic acid were assessed for any potential associations with the baseline growth rate of each cell line (Table 3). This analysis revealed strong inverse correlations between the initial baseline growth rate and responsiveness to hyaluronic acid. For example, phenotypes (viability, growth) of the most rapidly growing cell line CAL27 were robustly inhibited by the combination of HA with PD98059 or paclitaxel administration, while the phenotypes (viability, growth) of slowest growing cell line CCL-30 exhibited moderate increases. These data demonstrate an inverse (Pearson’s) correlation between baseline growth and responsiveness to HA, ranging from R = −0.27 to R = 0.883.

## 4. Discussion

The primary goal of this study was to investigate the effects of hyaluronic (alone and in combination with chemotherapeutic agents) using well-characterized oral cancer cell lines to determine any potential effects on cellular phenotypes, such as growth and viability. The results of this study revealed differing responses to both phenotypes, which may be inversely correlated with intrinsic characteristics, such as the baseline growth rate. This may suggest that the more rapidly growing cell lines are more responsive to combination therapy with hyaluronic acid; an important finding that may provide insights into the mechanisms responsible for these observations.

For example, one recent study demonstrated synergistic effects similar to those observed in this study with combination cisplatin–hyaluronic acid treatment in advanced metastatic ovarian cancer [19]. Another study also found that combination therapy involving hyaluronic acid exhibited synergistic and enhanced anti-tumor effects among aggressive breast cancers [20]. These data may suggest the results of this study may be consistent with other studies of combination therapies using hyaluronic acid with other aggressive and metastatic tumors.

In addition, these results may suggest that the extracellular matrix (ECM)-mediated responses may, in fact, play significant roles in tumor responsiveness to chemotherapy [21,22]. This may be due to the expression of hyaluronidases and matrix metalloproteinases, such as MMP-2 and MMP-9, among advanced and metastatic cancers that cleave ECM (including hyaluronic acid) and reduce cell-ECM contacts, adhesion, and the associated intracellular signaling [23,24]. In addition, this may also suggest that expression of MMP-2 and MMP-9 may serve as a biomarker or indicator to determine if combination therapy, including hyaluronic acid, may be indicated—although more research will be needed to make these determinations [25].

Although some previous studies have suggested that high expression of HA in both tumor and stromal cells from resected oral cancer patients may correlate with poor prognosis [11,14], these studies also confirm previous observations that proteolytic cleavage of HA by hyaluronidases and matrix metalloproteinases (MMPs) may trigger the invasion and metastasis via the receptor for hyaluronan (HA)-mediated motility (RHAMM) CD44 [18,25]. In fact, some evidence now suggests that CD44 acts as a receptor for proteolytically-modified HA and may be responsible for the modulation of intracellular signaling associated with increased proliferation and invasive capacity [17,26,27]. However, because the production of proteolytic enzymes with their cognate targets may be tightly regulated within these tumors, administration of intact, exogenous HA and the delivery of chemotherapeutic agents conjugated with HA has been sufficient in clinical studies to improve patient outcomes and reduce metastatic phenotypes in many cancer types [17,18,28,29,30].

## 5. Conclusions

Although much remains to be elucidated, these findings suggest that hyaluronic acid may, in fact, exert different effects in oral squamous cell carcinomas. Elucidation of the factors that more accurately determine oral cancer responsiveness, such as CD44, MMP-2, MMP-9, and hyaluronidase expression, may be needed to allow oral healthcare researchers and clinicians to more accurately assess and treat oral cancers with the most efficient and effective means of treatment that may include combination therapy with hyaluronic acid. Because this study revealed differential responses, which may be inversely correlated with intrinsic characteristics, such as baseline growth rate, the analysis and identification of those factors that correlate with responsiveness to combination therapy with hyaluronic acid may provide important insights into the mechanisms responsible for these observations.

## Figures and Tables

**Table 1:** Verification and cross-checking of cell lines.

Cell Line	Catalog Reference	STR % Match	Cell Type
HGF-1	CRL-2014	100%	Normal oral
SCC4	CRL-1624	92%	Oral squamous cell carcinoma
SCC9	CRL-1629	100%	Oral squamous cell carcinoma
SCC15	CRL-1623	94%	Oral squamous cell carcinoma
SCC25	CRL-1628	100%	Oral squamous cell carcinoma
CAL27	CRL-2095	93%	Oral squamous cell carcinoma
CCL-30	RPMI-2650	100%	Nasal septum carcinoma

**Table 2:** Establishment of Experimental Cell Cultures.

Cell Line	Confluence	Live Cell Count	Viability
SCC25 (oral cancer)	1.33 × 10^5^ cells/mL	1.23 × 10^5^ cells/mL	93%
SCC9 (oral cancer)	7.70 × 10^4^ cells/mL	7.01 × 10^4^ cells/mL	91%
SCC15 (oral cancer)	8.60 × 10^4^ cells/mL	7.65 × 10^4^ cells/mL	89%
CAL27 (oral cancer)	1.52 × 10^5^ cells/mL	1.35 × 10^5^ cells/mL	89%
CCL-30 (nasal cancer)	5.68 × 10^4^ cells/mL	4.66 × 10^4^ cells/mL	82%
SCC4 (oral cancer)	7.17 × 10^4^ cells/mL	5.31 × 10^4^ cells/mL	74%
HGF-1 (normal gingiva)	4.33 × 10^4^ cells/mL	3.81 × 10^4^ cells/mL	88%
			Ave: 86.2%

**Table 3:** Correlation of cellular phenotype responsiveness to hyaluronic acid (HA).

Cell Line	Baseline Growth (3d)	Viability PD98059+HA	Viability Paclitaxel+HA	Growth PD98059+HA	Growth Paclitaxel+HA
CAL27	1.4	−18	−4.8	−29.9	−13.6
SCC25	1.2	−6.9	−8.2	−30.9	−38.9
SCC15	0.8	16.1	5.9	27.7	4.1
SCC9	0.76	−1	16.1	−0.5	10.1
SCC4	0.58	1.8	−17.3	16.8	−9.7
CCL-30	0.55	11.1	10.7	18.11	17
Correlation		R = −0.80026	R = −0.27006	R = −0.8831	R = −0.68196
